# Three years of *Extreme Physiology & Medicine*

**DOI:** 10.1186/s13728-015-0033-x

**Published:** 2015-09-04

**Authors:** Michael P. W. Grocott, Hugh M. Montgomery

**Affiliations:** Integrative Physiology and Critical Illness Group, Division of Clinical and Experimental Science, Faculty of Medicine, University of Southampton, Southampton, UK; NIHR Respiratory Biomedical Research Unit, University Hospital Southampton NHS Foundation Trust, Southampton, UK; Anaesthesia and Critical Care Research Unit, CE.93 Mailpoint 24, E-Level Centre Block, University Hospitals Southampton NHS Foundation Trust, Tremona Road, Southampton, SO16 6YD UK; The Whittington Hospital NHS Foundation Trust, London, UK; University College Hospitals NIHR Comprehensive Biomedical Research Centre, University College London Hospitals Foundation Trust, London, UK

It is 3 years since *Extreme Physiology & Medicine* was launched as a high quality peer-reviewed open access journal focussing on integrative human physiology under conditions of physiological stress. Our third birthday seems an opportune moment to look back down the valley of our early days whilst looking forwards and upwards to peaks beyond.

In our welcoming editorial [1] we described the journal’s focus on ‘the integrative physiology of environmental, exertional and clinical stress in humans’. We emphasised how such study of human physiology could inform our understanding of *patho*physiology. This notion is gaining traction, in part given the increasing recognition that animal models of complex (patho)physiology may poorly represent that in humans [2]. For example, the notion that human-microbial challenge studies may offer the “ultimate animal model” for infectious diseases research has been proposed [3]. Our conviction of the translational value of integrative human physiology has only grown. Mike Tipton extended this concept further, describing eloquently how studies of the response to *combinations* of physiological stressors may offer even greater insights than single stressors alone [4].

When we launched *Extreme Physiology & Medicine*, we felt that few journals served the field of ‘extreme’ or ‘boundary’ physiology. This remains as true today as it was then. To ensure that *Extreme Physiology & Medicine* publishes relevant articles of the highest quality, our ambition was that submitted manuscripts would be handled by world leaders in their fields. In this regard, we are proud to have assembled a truly expert international board of editors who are at the cutting edge of their respective disciplines. It appears that we were correct in our estimation of unmet need: despite a rigorous reviewing process, we have published more than 80 articles in 3 years.

As well as publishing reports of scientific investigation, we also wished to create a journal which was ‘stimulating’ both to those with a passion for human integrative physiology and for a more general readership. We encourage submission of review articles relating to topics rarely covered elsewhere—such as the recent discussion of insights from metabolomics into the mitochondrial response to extreme environments, by O’Brien and colleagues [5]. Neither did we wish to be exclusive in our focus: we have reported new methods of arterialised blood sampling [6]; the demographics of performance in and the response to extreme endurance events [7]; a series on ‘moving in extreme environments’ [8]; and articles relating to (de)hydration [9], exposure to extreme heat and cold [10, 11], and severe hypoxia [12]—amongst many others. We have also commissioned ‘career perspectives’ from the Elder Statesmen/women of extreme physiology, encouraging them to reflect on ‘what made them who they are’ as much as ‘what they learned’—and all in their own voice [13].

*Extreme Physiology & Medicine* is an ‘Open Access’ journal: all content is readily accessible to all, free of charge, worldwide through the journal website (http://www.extremephysiolmed.com*) and* via SpringerLink (http://link.springer.com/journal/13728) as well as from a variety of digital archives (e.g. http://www.biomedcentral.com/libraries/archive). This approach is unique amongst specialist journals in this area, and improves both an article’s speed of dissemination and the scale of its “reach”, and therefore the likelihood that it will have impact and be cited. Such metrics are increasingly important to academia, and the value of such an approach for *Extreme Physiology & Medicine* is evident: >362,000 article accesses. All articles in *Extreme Physiology & Medicine* are indexed in PubMed and PubMed Central, as well as Scopus and Google Scholar, and the journal is included in the Directory of Open Access Journals.

Of course, the free-to-view, and reuse with proper citation, Open Access model shifts the cost of publication from the consumer to the producer, and thus attracts an article processing charge (£1370/$2145/€1745 per article in 2015). However, authors from the low-income countries (see http://www.biomedcentral.com/authors/oawaiverfund/) receive a full waiver, those whose parent institution is a member of the BioMed family pay no APCs directly, and those from BioMed supporter member institutions are eligible for a partial waiver or discount (see http://www.biomedcentral.com/libraries/membership). Increasingly, grant giving bodies and academic institutions demand open access publication and will meet the costs of publication.

Overall, then, how does our report card read? Hopefully ‘Good effort’ and ‘A fine start’ are reasonable. But ‘What could we do better’ is always on our minds. Please share with us your views and hopes for the journal along with any suggestions for improvements. We are confident that, with your support, the journal will continue to thrive and grow over the coming years. We look forward to receiving your article submissions and hope that you enjoy the unique contribution that we believe the journal makes to a rapidly developing and exciting field.
